# The effect of denosumab on pedicle screw fixation: a prospective 2-year longitudinal study using finite element analysis

**DOI:** 10.1186/s13018-021-02360-2

**Published:** 2021-03-26

**Authors:** Soji Tani, Koji Ishikawa, Yoshifumi Kudo, Koki Tsuchiya, Akira Matsuoka, Hiroshi Maruyama, Haruka Emori, Ryo Yamamura, Chikara Hayakawa, Masaya Sekimizu, Yusuke Oshita, Tomoyuki Ozawa, Toshiyuki Shirahata, Takashi Nagai, Tomoaki Toyone, Katsunori Inagaki

**Affiliations:** 1 Department of Orthopaedic Surgery, Showa University School of Medicine, 1-5-8 Hatanodai, Shinagawa, Tokyo, 142-8666 Japan; 2 Department of Orthopaedic Surgery, Yamanashi Red Cross Hospital, 6663-1 Funatsu Fujikawaguchiko-machi, Minamitsuru-gun, Yamanashi 401-0301 Japan; 3 Department of Orthopaedic Surgery, Showa University Northern Yokohama Hospital, 35-1, Chigasaki-Chuo Tsuzuki-ku, Yokohama, Kanagawa Japan; 4 Department of Orthopaedic Surgery, Tokyo Kyosai Hospital, 2-3-8, Nakameguro, Meguro, Tokyo, 153-0061 Japan; 5 Department of Orthopaedic Surgery, Showa University Koto Toyosu Hospital, 5-1-38 Toyosu, Koto-ku, Tokyo, 135-8577 Japan

**Keywords:** Denosumab, Finite element analysis, Pedicle screw, Loosening, Osteoporosis

## Abstract

**Background:**

Pedicle screw loosening is a major complication following spinal fixation associated with osteoporosis in elderly. However, denosumab is a promising treatment in patients with osteoporosis. The effect of denosumab on pedicle screw fixation is unknown. Therefore, we investigated whether denosumab treatment improves pedicle screw fixation in elderly patients with osteoporosis.

**Methods:**

This was a 2-year prospective open-label study. From February 2015 to January 2016, we included 21 patients with postmenopausal osteoporosis who received initial denosumab treatment. At baseline, 12 months, and 24 months, we measured volumetric bone mineral density (BMD) using quantitative computed tomography (QCT) and performed CT-based finite element analysis (FEA). Finite element models of L4 vertebrae were created to analyze the bone strength and screw fixation.

**Results:**

BMD increased with denosumab treatment. FEA revealed that both pullout strength of pedicle screws and compression force of the vertebra increased significantly at 12 and 24 months following denosumab treatment. Notably, pullout strength showed a stronger correlation with three-dimensional volumetric BMD around pedicle screw placement assessed by QCT (*r* = 0.83, at 24 months) than with two-dimensional areal BMD assessed by dual energy X-ray absorptiometry (*r* = 0.35, at 24 months).

**Conclusion:**

To our knowledge, this is the first study to reveal that denosumab treatment achieved strong pedicle screw fixation with an increase in BMD around the screw assessed by QCT and FEA; therefore, denosumab could be useful for osteoporosis treatment during spinal surgery in elderly patients with osteoporosis.

## Background

Pedicle screw (PS) fixation is widely used in spinal surgeries [1]. In an aging population, there is high incidence of degenerative spinal disorders, and some of these patients require surgery that includes PS fixation [2]. The number of patients presenting with spinal disorders that involve osteoporotic bone are also increasing [3, 4]. PS loosening is a major complication associated with osteoporosis [5]. Clinically, individuals who suffer from screw loosening experience pain and loss of function, and require revision surgery, all of which generate substantial personal and socioeconomic costs [6]. To overcome screw loosening, many studies have been conducted including osteoporosis treatment because there were some promising treatments related to screw fixation and bone union [7, 8].

Biomechanical studies have revealed factors affecting screw fixation, including mechanical properties, anatomical characteristics of the vertebrae, and insertion techniques [9, 10]. We previously reported that screw fixations were substantially affected by bone mineral density (BMD) around the PS [11]. Other studies have shown that osteoporosis treatment with bisphosphonates and teriparatide had positive effect on spinal fixation in both clinical and basic studies [12–15]. Although denosumab has been approved in various countries for treating patients with osteoporosis at high risk of fracture, including patients with spinal disorders [16], no clinical study has evaluated the use of denosumab for spinal surgery. Evidence shows that denosumab can be used for up to 10 years and has a wider indication than other drugs [17]. Therefore, there is a clinical need to better understand the effect of osteoporosis treatment during spinal surgery.

The aim of the present study was to determine the efficacy of denosumab treatment in elderly patients with osteoporosis for PS fixation by three-dimensional volumetric BMD (vBMD) using computed tomography (CT)-based finite element analysis (FEA) and perform a comprehensive analysis, including blood samples and two-dimensional areal BMD (aBMD) assessed by dual energy X-ray absorptiometry (DXA) to monitor the treatment efficacy.

## Methods

### Patients and study design

This study was a prospective, open-label study of patients with postmenopausal osteoporosis who were treated with denosumab for 24 months. Patients were included if they were over 60 years old and did not have histories of osteoporosis treatment and were excluded if they had (1) secondary osteoporosis, (2) poorly controlled thyroid disease, (3) active malignant tumors, (4) received or were scheduled to receive any surgery, and (5) had disease affecting musculoskeletal conditions such as Parkinson’s disease according to previous report [18, 19]. The sample size was calculated using G*Power software version 3.1.7 [20]. Effect size, alpha error, and beta error were set as 0.80, 0.05, and 0.20, respectively, based on a previous report [21]. The power analysis indicated that a total of 15 samples were needed. In consideration of potential discontinuation, 21 patients were included in this study from February 2015 to January 2016.

Study participants were treated with denosumab, 60 mg subcutaneously every 6 months for 24 months. They received daily supplementation of vitamin D and/or calcium for prophylaxis against hypocalcemia induced by denosumab [19]. Serum bone turnover markers (BTMs) were determined at baseline and every 6 months. DXA of the lumbar spine, femoral neck, and total hip, FEA, and quantitative CT (QCT) were performed at baseline, 12 months, and 24 months.

This study was conducted with the approval of the ethics committee of Yamanashi Red Cross Hospital and in accordance with the precepts of the Declaration of Helsinki. Written informed consent was obtained prior to any study-related procedures.

### Blood samples/bone turnover markers

We evaluated serum levels of albumin, calcium, phosphorus, alkaline phosphatase, estimated glomerular filtration rate, and intact parathyroid hormone at baseline and every 6 months according to our previous report [19]. The following BTMs were also measured at baseline and every 6 months: total N-terminal propeptide of type 1 procollagen (total P1NP; reference range in postmenopausal women, 26.4–98.2 μg/L; estimated using a total P1NP assay on an Elecsys automated analyzer; Roche Diagnostics, Switzerland) and tartrate-resistant acid phosphate type 5b (TRACP-5b; reference range in women, 120–420 mU/dL; estimated using Osteolink® TRACP-5b® test kit; DS Pharma Biomedical Co, Ltd., Osaka, Japan) [19].

### Two-dimensional areal bone mineral density

Areal BMD of the lumbar spine (L1–4) (LS-aBMD), femoral neck (FN-aBMD), and total hip (TH-aBMD) were measured using DXA (Hologic QDR series, Hologic Waltham, MA, USA). All DXA measurements were analyzed by a radiologist at a central site. The intra-observer coefficients of variation (%CVs) for the DXA in 20 osteoporosis patients were 0.95, 0.68, and 0.75 in the LS, FN, and TH, respectively, and the inter-observer %CVs were 0.85, 0.62, and 0.72, respectively.

### Three-dimensional volumetric bone mineral density

**Fig. 1:**
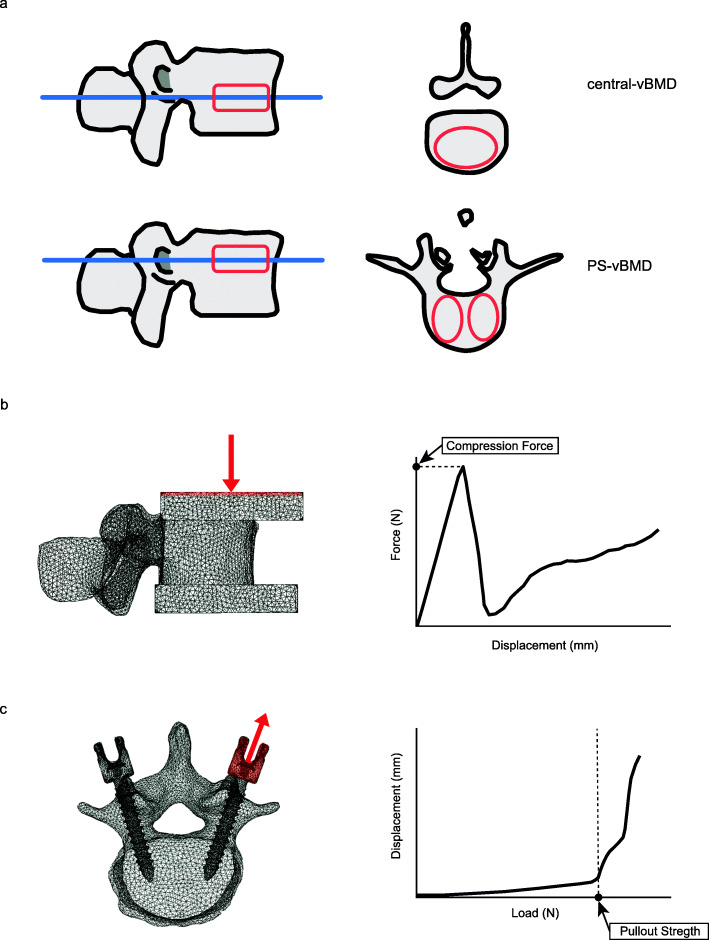
Standard template of central volumetric bone mineral density (central-vBMD) and pedicle screw volumetric bone mineral density (PS-vBMD) with oval-shaped region of interest (ROI). **a**. A finite element model of L4 vertebra with caps of poly(methyl-methacrylate) (PMMA) and a typical force-displacement curve illustrating compression force. **b** A finite element model of L4 vertebra with a pedicle screw and a typical load-displacement curve illustrating pullout strength. **c** Loading points (red) and displacement direction (red arrow)

Volumetric BMD at the lumbar spine (reference vertebra L4) was measured using a SOMATOM Definition AS+ multidetector-row CT scanner (Aquilion 16, Toshiba Medical System, Otawara, Japan) using predefined scanning conditions (X-ray energy, 120 kV; X-ray current, SD20; rotation speed, 0.5 s/rot; beampitch, 0.95). Using a semiquantitative method, we selected the L3 vertebra if the L4 vertebra had a grade 2 or 3 fracture [22]. We obtained 2-mm slices at 2-mm intervals. A calibration phantom (Mindways Software, Austin, TX, USA) was placed underneath the patients during CT. The central volumetric bone mineral density (vBMD) and pedicle screw-vBMD (PS-vBMD) of each lumbar vertebra were analyzed using QCT-Pro software v4.1.4 with the QCT-Pro Bone Investigational Toolkit v2.0 (BIT; Mindways Software, Austin, TX, USA). The region of interest (ROI) of central-vBMD and PS-BMD was defined manually as an oval-shaped, 7-mm-thick cursor according to our previous report [11]. Using QCT measurement, the ROI was established using both transverse and sagittal images (Fig. 1a). All procedures were conducted by three spine surgeons following to our previous report [11]. The intra-observer %CVs for the QCT were 3.21 in the central-vBMD, 5.15 in the PS-vBMD of the right side, and 5.25 in the PS-vBMD of the left side. The inter-observer %CVs were 3.52 in the central-vBMD, 5.35 in the PS-vBMD of the right side, and 5.68 in the PS-vBMD of the left side.

### FEA

The three-dimensional finite element (FE) models of the L4 vertebra were constructed from the CT data using Mechanical Finder software (version 9.0 extended edition; Research Center of Computational Mechanics, Tokyo, Japan). The modeling methods employed to develop these FE models and FEA were based on previous studies [23, 24]. The models were divided into 2-mm tetrahedral solid elements and 2-mm triangular plates [24]. Young’s modulus and thickness of each triangular plate were assigned values of 10 GP and 0.4-mm, respectively. To allow for bone heterogeneity, the BMD of each element was computed from the Hounsfield unit values. Young’s modulus and yield stress of each element were derived from a previous study by Keyak et al. and Poisson’s ratio of each element was set as 0.4 [25]. Each element was assumed to yield when its Drucker-Prager equivalent stress reached the element yield stress. Failure was defined as occurring when the minimum principal strain of an element was less than − 10,000 microstrain [24].

A compressive displacement was applied to the poly(methyl-methacrylate) (PMMA) cement cap at the cranial end of the vertebrae at ramped displacement increments of 0.01 mm. PMMA cement cap elements at the bottom of the vertebrae were encastered [26]. PMMA was assumed to have Young’s modulus of 2.5 GPa and Poisson’s ratio of 0.3. The predicted compression force (CF) was identified by a rapid decrease in the force-displacement curve (Fig. 1b) [26].

Each vertebra was implanted with the same PS (ZODIAC®-Spinal Fixation System, Alphatec) to assess the screw fixation. The lengths of the screws were 40 mm and the diameters were 7.5 mm. The FE models of the pedicle screws were developed from micro-CT data and were divided into 1-mm tetrahedral solid element. PSs were assumed to have the material properties of titanium alloy with Young’s modulus of 110 GPa, yield stress of 900 MPa, and Poisson’s ratio of 0.28. PSs were inserted into the vertebral bodies along the anatomical axis of the pedicle and parallel to the vertebral end plate, using Weinstein’s technique [27]. An incremental tensile loading rate of 20 Newton (N)/step was applied to the left side of the screw head, and construction was full fixation in all directions at the surface of the superior and inferior vertebral endplates. Under tensile loading conditions in the axis of the screw, the pullout strength (POS) was defined as the load of the flexion point on the load-displacement curve just before an abrupt increase in displacement [23] (Fig. 1c). To simulate peri-implant bone behaviors, the numbers of yield and failure elements when 600.0 N was loaded were evaluated. All FEA data were obtained from the Mechanical Finder software.

All procedures were conducted by three spine surgeons. The reproducibility of the FEA analyses was calculated using four repeated analysis, each with visual matching, from the CT data sets of six participants without visual artifacts. The intra-observer %CVs for the FEA were 3.56 and 5.15 in the CF and POS, respectively. The inter-observer %CVs were 5.52 and 6.85 in the CF and POS, respectively.

### Statistical analysis

All statistical analyses were performed by using nonparametric tests because of the small sample size [28]. Results were expressed as median with interquartile range (IQR). Changes in variable values from baseline measurements up to 24 months were tested for statistical significance using the Wilcoxon signed-rank test and Friedman’s test. The Holm-Bonferroni method was used for post-hoc corrections. Spearman’s correlation analysis was used to evaluate correlations between the values of FEA and BMD measurements. All statistical tests were two-tailed, and *P* values < 0.05 were considered statistically significant. All statistical analyses were performed with JMP® version 13 software (SAS Institute Inc., Cary, NC, USA) and EZR (Saitama Medical Center, Jichi Medical University, Saitama, Japan), a graphic user interface for R (R Foundation for Statistical Computing, Vienna, Austria) [29].

## Results

### Baseline patient characteristics

**Fig. 2:**
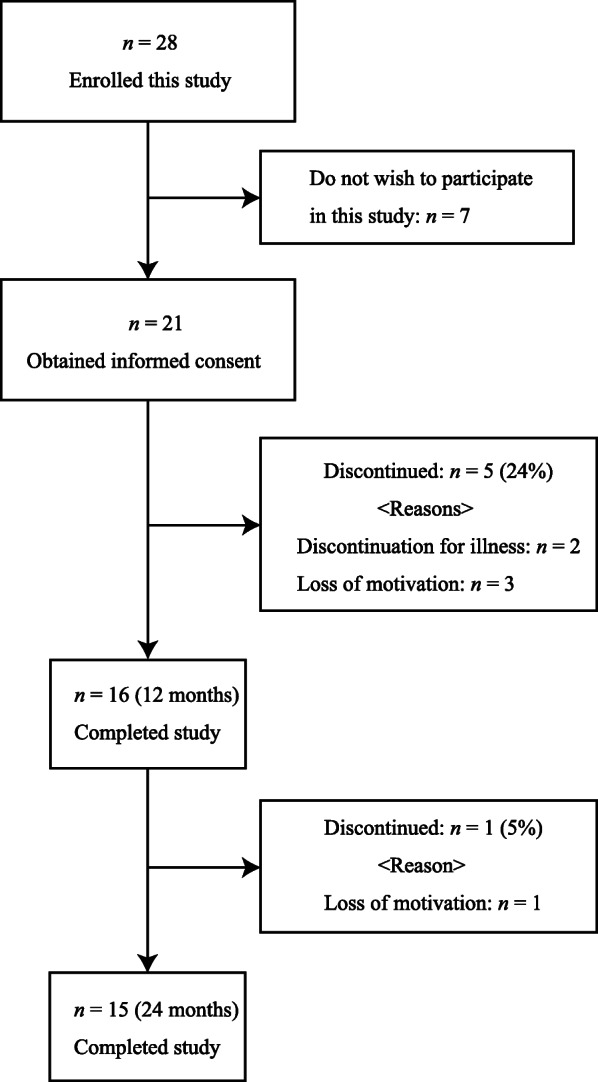
Study flow diagram

We enrolled 28 patients with postmenopausal osteoporosis, of which 21 provided informed consent and participated in the study protocol. Five subjects dropped out because of illness (*n* = 2) or loss of motivation (*n* = 3). Hence, 16 subjects completed the study for 12 months. Finally, 15 subjects completed the study for 24 months because one subject failed to complete the study at 18 months (Fig. 2). Baseline characteristics of these patients are given in Table 1. The median age was 67.0 years, and the body mass index was 23.0 kg/m^2^. All blood sample levels were within normal ranges, except TRACP-5b.

**Table 1:** Baseline patient characteristics

	*n* = 15
Age (years)	67.0 (65.0/75.5)
BMI (kg/m^2^)	23.0 (20.7/25.7)
Fracture, *n* (%)	9.0 (60.0)
History of family fracture, *n* (%)	1.0 (7.1)
Smoking, *n* (%)	0 (0)
Alcohol, *n* (%)	1.0 (6.7)
Albumin (mg/dL)	4.4 (4.3/4.4)
Calcium (mg/dL)	9.3 (9.2/9.6)
Phosphate (mg/dL)	3.6 (3.3/3.8)
Alkaline phosphatase (U/L)	301.0 (288.5/336.0)
eGFR (mL/min)	69.0 (62.0/90.0)
intact PTH (pg/mL)	35.0 (26.0/63.5)
Bone turnover markers	
total P1NP (μg/dL)	53.50 (40.00/91.60)
TRACP-5b (mU/dL)	412.00 (385.50/581.50)
Areal BMD (g/cm^2^) measured by DXA	
Lumbar spine	0.70 (0.66/0.77)
Femoral neck	0.56 (0.49/0.59)
Total hip	0.70 (0.63/0.74)
Volumetric BMD (mg/cm^3^) measured by QCT	
Central-vBMD	56.90 (49.22/70.27)
Right side of PS-vBMD	59.48 (52.94/82.25)
Left side of PS-vBMD	60.33 (53.67/81.03)

Data are expressed as median (Q1/Q3)

*BMI* body mass index, *eGFR* estimate glomerular filtration, *PTH* parathyroid hormone, *P1NP* N-terminal propeptide of type 1 procollagen, *TRACP*-*5b* tartrate-resistant acid phosphatase 5b, *BMD* bone mineral density, *DXA* dual energy X-ray absorptiometry, *QCT* quantitative computed tomography, *PS*-*vBMD* pedicle screw volumetric bone mineral density

### Bone turnover makers

**Fig. 3:**
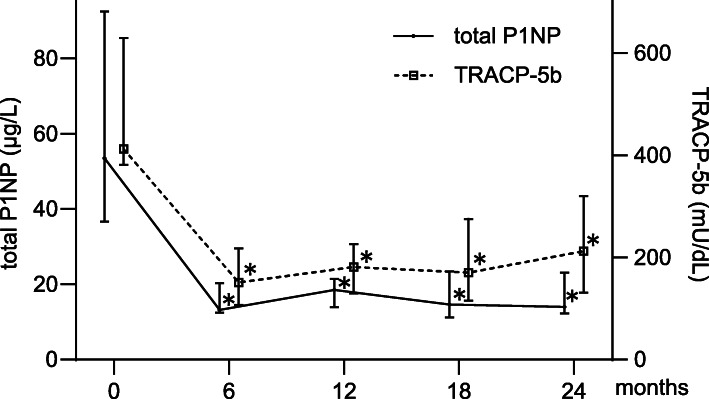
Longitudinal changes in bone turnover markers. Median changes in total type 1 N-terminal propeptide (total P1NP) and tartrate-resistant acid phosphate type 5b (TRACP-5b) levels during denosumab treatment (*P < 0.01 versus baseline; Wilcoxon signed-rank test). Data are shown as median (interquartile range)

Changes in the total P1NP and TRACP-5b levels are shown in Fig. 3. The total P1NP and TRACP-5b levels decreased at 6 months and remained suppressed until 24 months of treatment. Significant differences were found in the total P1NP and TRACP-5b levels at 6, 12, 18, and 24 months compared with the baseline (all *P* < 0.01).

### aBMD measured by DXA

**Fig. 4:**
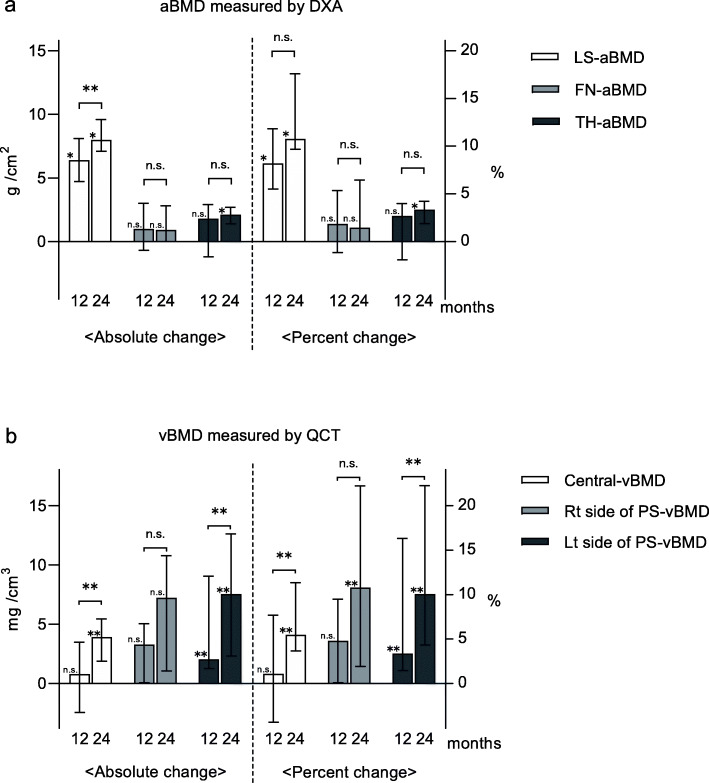
Absolute and percent changes in lumbar spine areal bone mineral density (LS-aBMD), femoral neck aBMD (FN-aBMD), and total hip aBMD (TH-aBMD). **a** Absolute and percent changes in volumetric BMD (vBMD) at the center and bilateral side of the pedicle screws. **b** (*P < 0.01, **P < 0.05, not significant [n.s.]; Friedman’s test). Data are expressed as medians (interquartile ranges)

Changes in areal bone mineral density (aBMD) from baseline are shown in Fig. 4a. The absolute and percent changes in LS-aBMD were significantly greater after 12 and 24 months of treatment (all *P* < 0.01). The absolute and percent changes in FN-aBMD from baseline were not significant. The absolute and percent changes in TH-aBMD at 24 months were significantly greater than at baseline (both *P* < 0.01).

### vBMD measured by QCT

Changes in vBMD from baseline are shown in Fig. 4b. The absolute and percent changes in central-vBMD at 24 months were significantly greater than at baseline (both *P* < 0.05). The absolute and percent changes in the left side of PS-vBMD significantly increased at 12 and 24 months (all *P* < 0.05).

### FEA

**Fig. 5:**
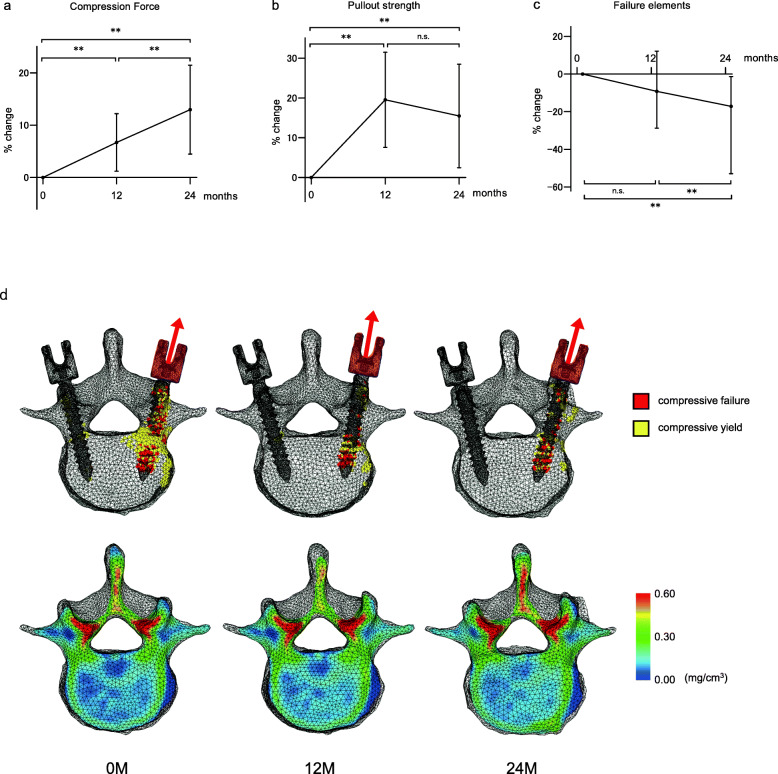
Longitudinal changes in compression force (**a**), pullout strength (**b**), and number of failure and yield elements when 600 N was loaded (**c**). Longitudinal changes in compressive failure and yield element distribution when 600 N was loaded at the left side of the pedicle screw, and distribution of bone mineral density (**d**) (**P < 0.05, not significant [n.s.] versus baseline; Friedman’s test). Data are expressed as medians (interquartile ranges)

FEA showed increases in both CF and POS (Fig. 5a, b), significantly increasing following 12 and 24 months of treatment. The median CF at baseline, 12 months, and 24 months were 3817.8 N, 3898.3 N, and 4190.2 N, respectively (both *P* < 0.05, versus baseline, 12 months, and 24 months). The median POS at baseline, 12 months, and 24 months were 400.0 N, 480.0 N, and 440.0 N, respectively (both *P* < 0.05, versus baseline, 12 months, and 24 months). The numbers of yield and failure elements when 600.0 N was loaded decreased at 24 months (*P* < 0.05 versus baseline) (Fig. 5c). The yield and failure elements (compressive yield and failure) at the region of bone destruction and bone mineral distribution cross-section of the axial plane at the PS level are shown in Fig. 5d. Many failure elements around the PS before treatment diminished during denosumab treatment.

### Correlation between FEA and BMD

Correlation analysis between BMD measurement and the value of FEA were performed to evaluate how FEA measurement changes with BMD. The values of CF and POS exhibited significant correlations with each BMD values (Table 2). Interestingly, CF and POS showed stronger correlations with central-vBMD and PS-vBMD, respectively.

**Table 2:** Correlation coefficients between the values of CF or POS and LS-aBMD and central-vBMD and PS-vBMD

	LS-aBMD		Central-vBMD		PS-vBMD	
	*r*	*P*	*r*	*P*	*r*	*P*
CF 0M	0.38	0.16	0.49	0.08	0.39	0.08
CF 12M	0.29	0.29	0.64	< 0.05	0.60	< 0.05
CF 24M	0.34	0.22	0.78	< 0.01	0.67	< 0.01
POS 0M	0.58	< 0.05	0.45	0.16	0.39	0.19
POS 12M	0.34	0.21	0.54	< 0.05	0.58	< 0.05
POS 24M	0.35	0.20	0.62	< 0.05	0.83	< 0.01

*0M* baseline, *12M* 12 months, *24M* 24 months, *LS*-*aBMD* lumbar spine areal bone mineral density, *central*-*vBMD* central volumetric bone mineral density, *PS*-*vBMD* pedicle screw volumetric bone mineral density of the left side, *CF* compression force, *POS* pullout strength, *r* correlation coefficient, *P P* value, *n*.*s*. not significant

No relationship was found between aBMD and the numbers of yield and failure elements; however, significant correlations were exhibited between vBMD and the number of yield and failure elements (Table 3).

**Table 3:** Correlation coefficients between the number of failure elements and LS-aBMD, central-vBMD, and PS-vBMD

	LS-aBMD		Central-vBMD		PS-vBMD	
	*r*	*P*	*r*	*P*	*r*	*P*
Number of failure elements 0M	− 0.35	0.20	− 0.61	< 0.01	− 0.83	< 0.01
12M	− 0.50	0.06	− 0.65	< 0.01	− 0.73	< 0.01
24M	− 0.47	0.08	− 0.55	< 0.05	− 0.61	< 0.05

*0M* baseline, *12M* 12 months, *24M* 24 months, *LS*-*aBMD* lumbar spine areal bone mineral density, *central*-*vBMD* central volumetric bone mineral density, *PS*-*vBMD* pedicle screw volumetric bone mineral density of the left side, *CF* compression force, *r* correlation coefficient, *P P* value, *n*.*s*. not significant

## Discussion

In the present study, we determined the effect of denosumab treatment using FEA. CF and POS increased following 2 years of denosumab treatment. To the best of our knowledge, no previous studies have reported the effect of denosumab treatment during spinal instrumentation. We also found that the failure elements elicited by the pullout force decreased. Taken together, our results provided the first insight into the possible effects of denosumab treatment with respect to the integrity of screw fixation.

In patients with postmenopausal osteoporosis, estrogen deficiency increases the numbers and activity of osteoclasts through a mechanism driven by receptor activator of nuclear factor-κB ligand (RANKL). Denosumab is a fully human monoclonal antibody against RANKL that reduces osteoclast numbers and activity, resulting in decreased bone resorption. Receptor activator of nuclear factor-κB (RANK) and its corresponding ligand RANKL are important regulators of osteoclast activity and bone resorption and are associated with osteolysis around implants [30]. Bernhardsson et al. [31] showed that denosumab treatment improved implant fixation in rats; nevertheless, there have been no studies of denosumab treatment improving PS fixation in patients. Crotti et al. [32] reported that RANKL/osteoprotegerin ratio was greater around peri-implant tissues of patients with implant failure. Another study suggested that denosumab was effective in a rat model of prosthetic loosening [33]. These findings suggest that denosumab helps prevent peri-implant failure (including PS loosening) by inhibiting the RANKL-RANK pathway.

In an aging population, there is a high incidence of degenerative spinal disorders, and the numbers of spinal fusion surgeries have increased significantly [1]. BMD is a key factor in determining long-term outcomes of patients with spinal surgery, and low BMD has a strong relationship with screw loosening [5]. DXA has been used to evaluate osteoporosis treatment; however, estimation of the changes in bone strength remains challenging. Areal BMD measured by DXA could be overestimated by factors that are common in elderly people, including prevalence of osteophyte formation, degeneration of the facet joint, aortic calcification, and sclerosis of the intervertebral discs [34]. Recent studies have shown that patient-specific FE methods are convenient and reproducible analytic methods because FEA predicts bone strength and fracture sites accurately [24, 26]. Tawara et al. showed that osteoporosis treatment decreased yield and failure elements and increased vertebral strength by FEA similar to in vivo [35]. In the present study, decreased numbers of yield and failure elements were observed following denosumab treatment. The results might suggest that resistance to micro-motion, which led to PS loosening, is increasing following osteoporosis treatment.

Two-dimensional BMD and PS fixation had been evaluated in previous studies [23, 36]. We previously reported that trabecular three-dimensional BMD around the PS (PS-vBMD) that is missed in a DXA measurement was an important parameter for screw insertion torque [11]. In the present study, PS-vBMD significantly increased by 10.1% at 24 months. We also investigated the relationship between each BMD measurement and POS to determine which area of the BMD is most affected, and we found that PS-vBMD had the strongest correlation with screw fixation, supporting the findings of our previous report [11]. In terms of vertebral strength, CF was significantly greater following denosumab treatment as in previous studies [21]. Interestingly, we observed that central-vBMD had stronger correlations with CF than other BMD measurements. Taken together, specific three-dimensional vBMD might be a high potency predictor of biomechanical analysis.

Denosumab treatment was reportedly more effective on the cortical bone than on the trabecular bone [37, 38]. Hirano et al. [10] reported that 60% of the POS of the PS depended on the pedicle that consisted primarily of cortical bone. Thus, the effect of the cortical bone might be one of the reasons why PS fixation was increased following denosumab treatment.

Given the increasing number of patients with osteoporosis, surgeons need to consider bone quality more carefully than ever. Although we have treatment options [12, 13], there is no standard medical approach to manage peri-operative patients with osteoporosis. Our novel insights suggested a valuable therapeutic option.

Several limitations of this study should be acknowledged. First, the goal of spinal instrumentation is bone fusion. Although we were unable to evaluate bone fusion, a previous study reported that denosumab did not interfere with fracture healing [39]. Furthermore, there is the possibility of increasing intervertebral fusion following treatment with bisphosphonate, an antiresorptive agent similar to denosumab [13]. At the least, rigid screw fixation following denosumab treatment could contribute to bone fusion. Second, even though previous studies have validated FEA [26, 40], it did not completely replicate the clinical situation. For instance, the screw was inserted until the last thread, and there was no contact between the screw head and the bone surface. Finally, in the present study, we could not set the true control group because denosumab treatment was only indicated to patient with osteoporosis (the Japan diagnostic criteria, [i] presence of a fracture in either the lumbar spine or the proximal femur, [ii] presence of another fragility fracture and a BMD below 80% of young adult mean, and [iii] BMD is equal to or below either 70% or − 2.5 SD of young adult mean) [41].

## Conclusion

In conclusion, this was the first study to show that denosumab treatment increased the screw fixation and vertebral strength, which correlated with specific three-dimensional BMD. Denosumab might represent a valuable therapeutic option for peri operative management of patients with osteoporosis. Patient-specific FEAs might be useful to evaluate the therapeutic effect of osteoporosis treatment during spinal surgery.

## Data Availability

All data and materials were collected from the electronic medical record system.

## Abbreviations

BMD: Bone mineral density
CT: Computed tomography
FEA: Finite element analysis
BTMs: Bone turnover markers
DXA: Dual energy X-ray absorptiometry
QCT: Quantitative computed tomography
total P1NP: Total N-terminal propeptide of type 1 procollagen
TRACP-5b: Tartrate-resistant acid phosphate type 5b
LS: Lumbar spine
FN: Femoral neck
TH: Total hip
aBMD: Areal bone mineral density
%CV: Coefficient of variation
vBMD: Volumetric bone mineral density
PS: Pedicle screw
PMMA: Poly(methyl-methacrylate)
FE: Finite element
ROI: Region of interest
CF: Compression force
N: Newton
POS: Pullout strength
RANKL: Receptor activator of nuclear factor-κB ligand
RANK: Receptor activator of nuclear factor-κB
